# Supplementary material for: Atomically Thin Amorphous Indium–Oxide Semiconductor Film Developed Using a Solution Process for High-Performance Oxide Transistors
Source: Nanomaterials (Basel). 2023 Sep 16;13(18):2568. doi: 10.3390/nano13182568 (PMC10536517; doi:10.3390/nano13182568)
Supplement: Supplementary file 1 [file nanomaterials-13-02568-s001.zip › nanomaterials-2593056-supplementary.pdf]

nanomaterials

Article

Atomically Thin Amorphous Indium–Oxide Semiconductor Film Developed Using a Solution Process for High-Performance Oxide Transistors

Jun-Hyeong Park, Won Park, Jeong-Hyeon Na, Jinuk Lee, Jun-Su Eun, Junhao Feng, Do-Kyung Kim *,† and Jin-Hyuk Bae *

School of Electronic and Electrical Engineering, Kyungpook National University, Daegu 41566, Republic of Korea; jeef1234@knu.ac.kr (J.-H.P.)
* Correspondence: kdk7362@knu.ac.kr (D.-K.K.); jhbae@ee.knu.ac.kr (J.-H.B.)
† Present address: LG Display, Paju 10845, Republic of Korea.

Supplementary Materials

Figure Captions

Figure S1. Proposed chemical reaction mechanism of InO_x by water etchant-based photopatterning method.

Figure S2. GIXRD spectra of InO_x thin films with various solution molarities.

Figure S3. In, O, and Si element mappings of (a) InO_x-5, (b) InO_x-7, (c) InO_x-9, and (d) InO_x-11 deposited on SiO_2 dielectric.

Figure S4. $I_D^{\frac{1}{2}} - V_G$ curves and linear fitting of (a) InO_x-5, (b) InO_x-7, (c) InO_x-9, and (d) InO_x-11.

Figure S5. (a) Schematic illustration of fabricated TFT arrays and measurement points (red box) for statistical evaluation. (b) Distribution of V_T (top) and μ_{FE} (bottom) of three InO_x-7 samples. (c) Statistical data of V_T (left) and μ_{FE} (right) for three InO_x-7 samples (10 points per sample).

Table Captions

Table S1. Summary of recently reported solution-processed oxide TFTs with SiO_2 dielectrics and this work. The data are used in Figure 5.

Figures

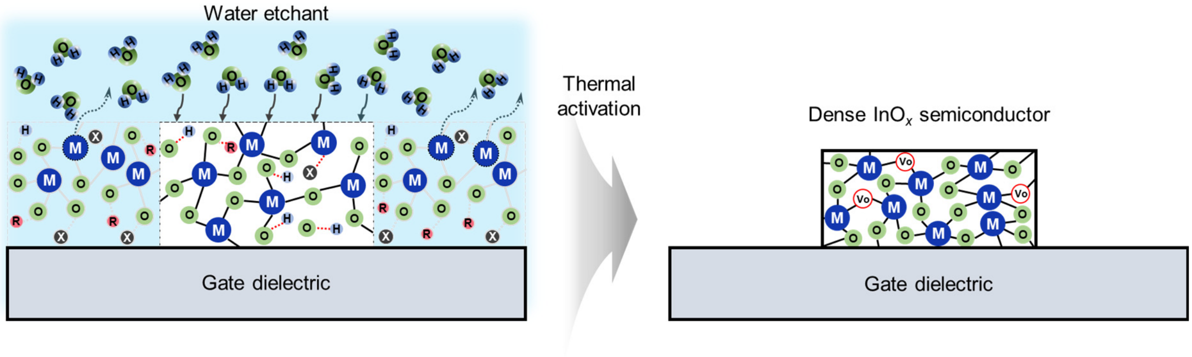

Figure S1. Proposed chemical reaction mechanism of InO_x by water etchant-based photopatterning method.

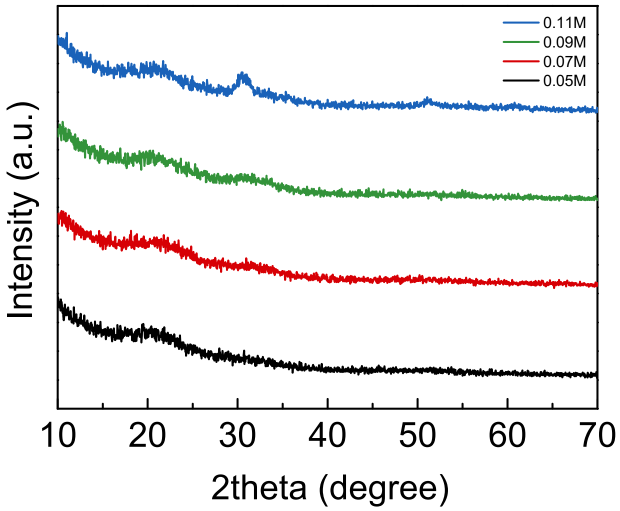

Figure S2. GIXRD spectra of InO_x thin films with various solution molarities.

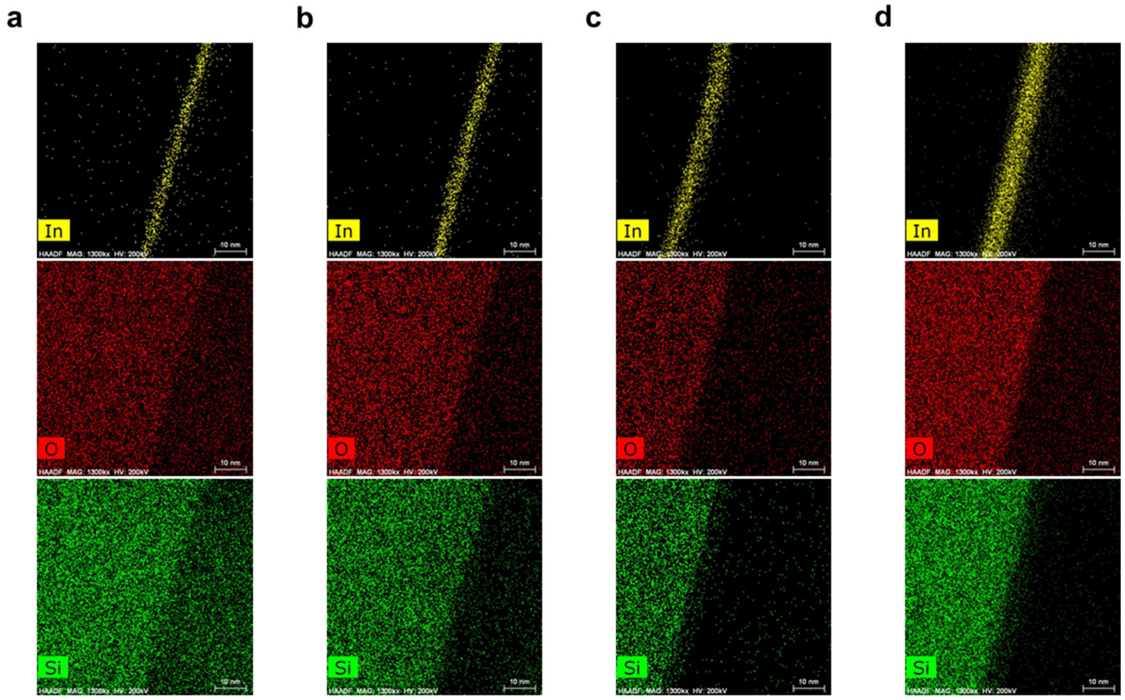

Figure S3. In, O, and Si element mappings of (a) InO_x-5, (b) InO_x-7, (c) InO_x-9, and (d) InO_x-11 deposited on SiO_2 dielectric.

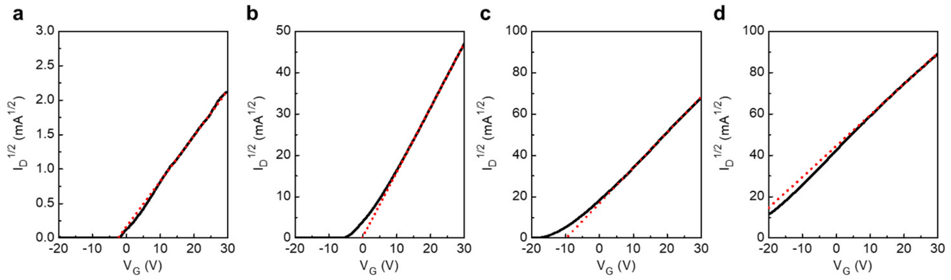

Figure S4. $I_D^{\frac{1}{2}} - V_G$ curves and linear fitting of (a) InO_x-5, (b) InO_x-7, (c) InO_x-9, and (d) InO_x-11.

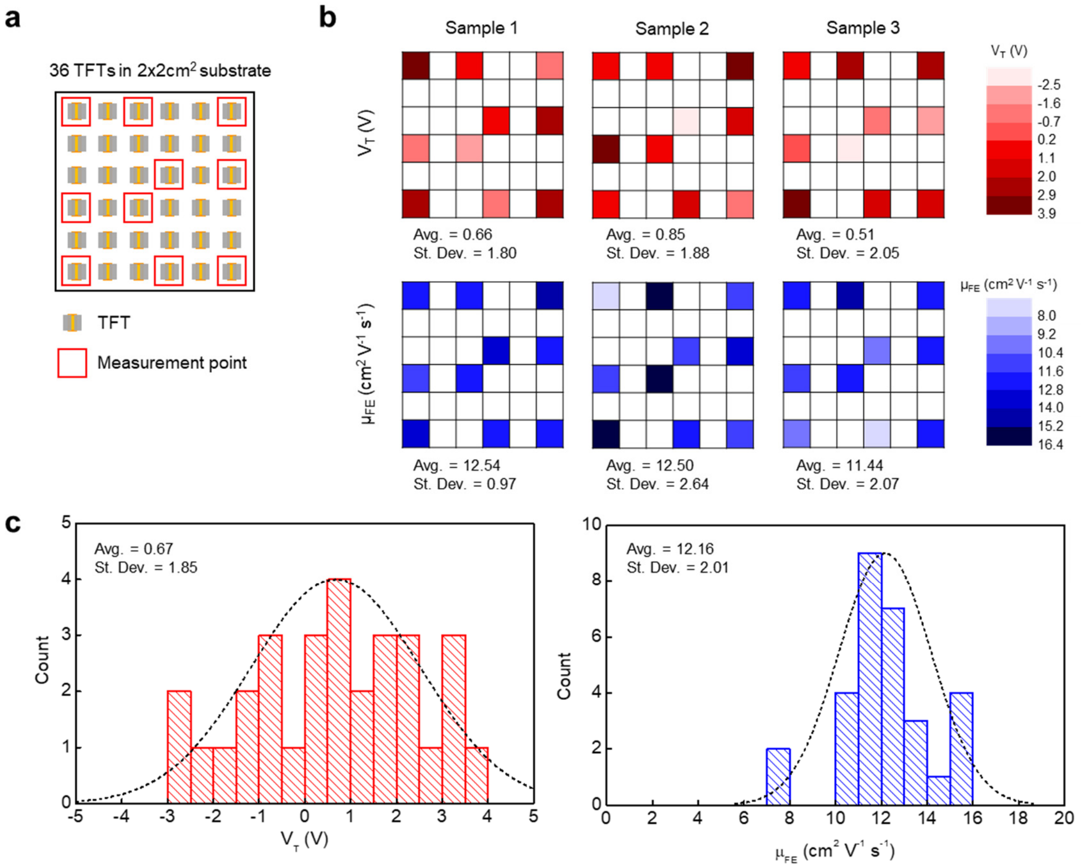

Figure S5. (a) Schematic illustration of fabricated TFT arrays and measurement points (red box) for statistical evaluation. (b) Distribution of V_T (top) and μ_{FE} (bottom) of three InO_x-7 samples. (c) Statistical data of V_T (left) and μ_{FE} (right) for three InO_x-7 samples (10 points per sample).

Tables

Table S1. Summary of recently reported solution-processed oxide TFTs with SiO_2 dielectrics and this work. The data are used in Figure 5.

Year	Semiconductor	T (℃)	V_T (V)	μ_{FE} (cm^2 V^{-1} s^{-1})	$I_{on/off}$	Ref.
2013	ZnSnO	500	15.00	1.99	2.4×10^8	[1]
2014	ZnSnO	400	0.04	4.84	$\sim10^7$	[2]
2015	InO_x	200	9.60	1.44	$\sim10^6$	[3]
2015	InO_x	225	9.10	3.15	$\sim10^6$	[3]
2015	InO_x	250	-11.20	6.11	$\sim10^4$	[3]
2015	InO_x	300	-26.70	15.45	$\sim10^4$	[3]
2015	InO_x	200	-1.60	5.52	1.7×10^5	[4]
2015	InO_x	250	5.80	3.84	6.0×10^7	[4]
2015	InO_x	250	8.46	8.84	$\sim10^7$	[5]
2015	InO_x	250	20.30	0.36	1.4×10^6	[6]
2015	InO_x	300	13.60	1.24	1.9×10^7	[6]
2015	InO_x	300	12.70	3.08	$\sim10^8$	[6]
2015	InO_x	300	6.20	4.61	$3.3\text{x }10^4$	[6]
2015	InO_x	350	3.20	4.67	1.1×10^3	[6]
2015	QSL-III	180	-8.3	46.3	$\sim10^5$	[7]
2016	InGaO	350	1.01	2.18	$\sim10^7$	[8]
2016	InGaO	500	1.64	1.99	$\sim10^7$	[8]
2017	LiZnO	500	5.80	3.80	2.2×10^7	[9]
2017	MgInO	500	2.84	13.77	2.4×10^7	[10]
2017	NbZnSnO	430	7.00	7.41	$\sim10^8$	[11]

2018	ZnSnO	500	6.88	9.13	8.8×10^{6}	[12]
2018	ZnSnO	500	3.85	8.74	4.3×10^{8}	[12]
2018	ZnSnO	260	9.30	3.35	1.1×10^{8}	[12]
2018	InZnO	280	1.41	7.82	~10^{7}	[13]
2018	GaCdO	450	-6.80	5.1	2.1×10^{7}	[14]
2018	InSnZnO/InGaZnO	450	-3.40	38.09	3.6×10^{8}	[15]
2018	ZnSnO	500	1.75	3.70	9.5×10^{6}	[16]
2019	ZnSnO	300	10.60	0.16	~10^{5}	[17]
2019	ZnSnO	400	3.07	3.00	~10^{6}	[17]
2019	ZnSnO	500	1.03	5.90	~10^{7}	[17]
2019	ZnSnO	600	-5.30	6.40	~10^{3}	[17]
2019	InSmO	350	2.14	21.51	1.1×10^{8}	[18]
2019	InGaZnO	300	6.00	2.50	3.2×10^{6}	[19]
2020	InGnZnO	350	3.70	1.27	3.3×10^{7}	[20]
2021	InGnZnO	300	0.20	8.50	~10^{7}	[21]
2021	InO_x/ZnO	210	7.05	17.84	~10^{5}	[22]
2022	ZnSnO	500	-0.95	2.41	3.9×10^{6}	[23]
2022	InPrO	350	4.8	17.03	2.2×10^{6}	[24]
	InO_x	300	-0.60	13.71	1.4×10^{10}	This work
	InO_x	300	1.97	12.06	1.1×10^{11}	This work
	InO_x	300	-0.01	15.41	7.4×10^{9}	This work
	InO_x	300	3.38	11.64	5.4×10^{9}	This work
	InO_x	300	-1.18	10.04	1.4×10^{8}	This work
	InO_x	300	-0.19	12.88	4.4×10^{9}	This work
	InO_x	300	3.42	11.96	9.8×10^{8}	This work
	InO_x	300	2.20	14.89	1.9×10^{8}	This work
	InO_x	300	3.38	11.66	1.8×10^{10}	This work
	InO_x	300	2.01	12.45	5.4×10^{10}	This work
